# Identification of Flavonoids in the Leaves of *Eranthis longistipitata* (Ranunculaceae) by Liquid Chromatography with High-Resolution Mass Spectrometry (LC-HRMS)

**DOI:** 10.3390/plants10102146

**Published:** 2021-10-10

**Authors:** Vera A. Kostikova, Alexander A. Chernonosov, Alexander A. Kuznetsov, Natalia V. Petrova, Denis A. Krivenko, Olga A. Chernysheva, Wei Wang, Andrey S. Erst

**Affiliations:** 1 Central Siberian Botanical Garden, Siberian Branch of Russian Academy of Sciences, 630090 Novosibirsk, Russia; 2 Laboratory Herbarium (TK), Tomsk State University, 634050 Tomsk, Russia; ys.tsu@mail.ru; 3 Institute of Chemical Biology and Fundamental Medicine, Siberian Branch of Russian Academy of Sciences, 630090 Novosibirsk, Russia; alexander.chernonosov@niboch.nsc.ru; 4 Komarov Botanical Institute, Russian Academy of Sciences, 197376 St. Petersburg, Russia; npetrova@binran.ru; 5 Siberian Institute of Plant Physiology & Biochemistry, Siberian Branch of Russian Academy of Sciences, 664033 Irkutsk, Russia; krivenko.irk@gmail.com (D.A.K.); helga8408@mail.ru (O.A.C.); 6 Institute of Botany, Chinese Academy of Sciences, Beijing 100093, China; wangwei1127@ibcas.ac.cn

**Keywords:** *Eranthis longistipitata*, LC-HRMS, flavonoid, chemical composition, extracts

## Abstract

*Eranthis longistipitata* Regel is an endemic plant of Central Asia. The flavonoid profile of *E. longistipitata* leaves was studied by mass spectrometry for the first time (natural populations of Kyrgyzstan and Uzbekistan, in 70% aqueous–ethanol extracts by liquid chromatography coupled with high-resolution mass spectrometry). Mass spectrometry revealed 18 flavonoid compounds. Flavonols featured the highest diversity, and 10 such substances were identified: 2 free aglycones (quercetin and kaempferol), 6 quercetin glycosides (peltatoside, hyperoside, reynoutrin, quercetin 3-sambubioside, rutin, and isoquercitrin), and 2 kaempferol glycosides (juglalin and trifolin). Two flavans (cianidanol and auriculoside), two hydroxyflavanones (6-methoxytaxifolin and aromadendrin), and one C-glycoside flavone—carlinoside—were identified. Dihydroxychalcones aspalathin, phloridzin, and phloretin were found too. Levels of rutin, quercetin, kaempferol, and hyperoside were confirmed by means of standards and high-performance liquid chromatography. Rutin concentration was the highest among all other identified flavonoid compounds: in the leaf samples from Kyrgyzstan, it ranged from 2.46 to 3.20 mg/g, and in those from Uzbekistan, from 1.50 to 3.01 mg/g. The diversity of flavonoid compounds in *E. longistipitata* leaves is probably due to external ecological and geographic factors and adaptive mechanisms.

## 1. Introduction

The genus *Eranthis* Salisb. belongs to the tribe Cimicifugeae of the family Ranunculaceae Juss., with 13 species occurring on a territory covering a part of Europe and Asia [1,2]. Representatives of this genus are perennial herbs with tuberous rhizomes or tubers and basal long petiolate leaves with the blades divided into several or many palmate segments that are undivided or lobate; unbranched scapes carry a solitary bisexual actinomorphic flower supported by three verticillate leaf-like bracts forming involucrate leaves; there are (4–)5–8 yellow, white or pink, petaloid sepals as well as 5–10(–15) yellow or white, petaloid, or slightly concave tubular petals, shorter than sepals, sometimes with pseudonectaries; nectaries are located inside the tubular petal; there are >10 stamens and 3–10 follicles with several smooth seeds in each fruitlet [3,4]. According M. Tamura [5], on the basis of morphology, the genus has been divided into two sections: *Eranthis* (*E. bulgarica* (Stef.) Stef., *E. cilicica* Scott & Kotschy, *E. hyemalis* (L.) Salisb., *E. iranica* Rukšāns & Zetterl., and *E. longistipitata* Regel.) and *Shibateranthis* (Nakai) Tamura (*E. albiflora* Franch., *E. byunsanensis* B.Y.Sun, *E. lobulata* W.T.Wang, *E. pinnatifida* Maxim., *E. pungdoensis* B.U.Oh, *E. sibirica* DC., *E. stellata* Maxim., and *E. tanhoensis* Erst). The section *Eranthis* is characterized by tuberous rhizomes or tubers, yellow sepals, and an emarginate or slightly bilobate upper petal apex without pseudonectaries. Representatives of the sect. *Shibateranthis* have tubers, white sepals, and a bilobate or forked petal apex, sometimes with pseudonectaries [6]. Phylogenetic studies support the subdivision of the genus into these sections [2,7].

Even though some species are widely distributed (*E. hyemalis* and *E. stellata*), most species of the genus are narrow endemics and have a small geographic range, which causes difficulties with comprehensive investigation into representatives of this group of plants. Nonetheless, a comprehensive analysis of the genus *Eranthis*—as a species-poor model group of the Ranunculaceae family—provides an opportunity for obtaining data that can be used in many areas of biology and related sciences. For example, information about the chemical composition and biological/therapeutic effects of *Eranthis* species is fragmentary. It is known that *Eranthis* species are used to treat urolithiasis and diuresis [8], and an ethanol extract from *E*. *hyemalis* roots has an anti-inflammatory activity [9]. Triterpene glycosides of cycloartan and oleanan series and triterpene saponins have been discovered in the tubers of *E*. *cilicica*. These substances are toxic to human promyelocytic leukemia (HL-60) cells [10]. Antioxidant properties of chromones isolated from *E. cilicica* tubers have been documented [11]. Chromones and lectins with anticancer, insecticidal, antifungal, and antiviral effects have been found in the tubers of *E. hyemalis*. [12,13].

Data on the profile and levels of polyphenols in plants of the genus *Eranthis* are still scarce, even though polyphenols are some of the most common representatives of secondary metabolites in plant tissues. Due to their strong biological activity, plant polyphenols are successfully used in various industries, medicine, and pharmacology as substances with antioxidant, neuroregulatory, capillary-strengthening, immunomodulatory, anticancer, and other effects [14,15]. Recent research indicates that one of the most common families of polyphenols—flavonoids—may have an inhibitory activity against coronaviruses including severe acute respiratory syndrome coronavirus 2 (SARS-CoV-2), which caused the current pandemic designated as COVID-19 [16]. In addition, polyphenols are widely employed in chemotaxonomic and phylogenetic research, owing to their high prevalence among plants, structural diversity, and chemical stability [17]. Polyphenols from the leaves of Ranunculaceae plants hold promise as chemotaxonomic markers [1,8].

We have previously revealed the specificity of the profile of polyphenols in the leaves of *Eranthis* plants, section *Shibateranthis* [1]. In that study, the set of polyphenols in the leaves of *E. sibirica*, *E. stellata*, and *E. tanhoensis* was determined by high-performance liquid chromatography [1]. Elsewhere, a difference in the ratio of flavonoids to phenolcarboxylic acids (in terms of concentration) has been revealed among these plant species: *E. sibirica* differs from the other two species [18].

There are no published data on the set and levels of polyphenols in plants of the section *Eranthis*, including *E. longistipitata*. Perhaps this is due to the inaccessibility of the plant material for collection: *E. longistipitata* is an endemic plant of Central Asia that grows on steppe slopes of foothills, and its populations are few. Collecting the material is also problematic because *E. longistipitata*—as with all ephemeroids—has a short growth season, and after flowering in early spring, the aboveground part of the plant dies off completely. Recent studies successfully showed in vitro reproduction of representatives of this genus, including *E. longistipitata* [19]. This advancement should protect natural populations from extinction and enable practical use of this plant.

This study aimed at investigating the pattern and concentrations of flavonoids in the leaves of *E. longistipitata* from natural populations of Kyrgyzstan and Uzbekistan by liquid chromatography coupled with high-resolution mass spectrometry (LC-HRMS), which requires only a small number of samples and, therefore, is promising for the investigation of plants of this genus.

## 2. Results and Discussion

**Figure 1:**
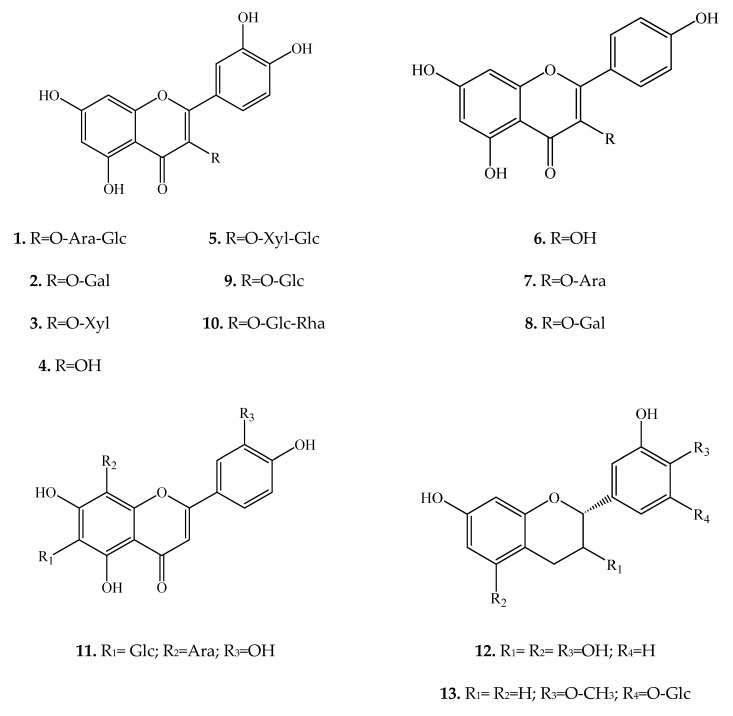

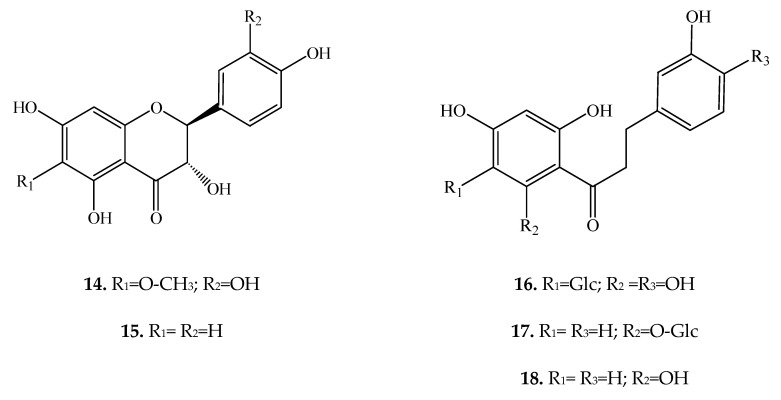
Structures of compounds **1**–**18** in *E. longistipitata*. Ara: arabinopyranose; Gal: galactopyranose; Xyl: xylopyranose; Glc: glucopyranose; Rha: rhamnopyranose.

The profile of flavonoids in *E. longistipitata* leaves was studied by mass spectrometry for the first time (by LC-HRMS in 70% aqueous-ethanol extracts; Table 1, Figure 1). For compound identification, the mass spectrometry (MS) data obtained in both positive and negative electrospray ionization (ESI-/ESI+) modes were compared with data from databases mzCloud and ChemSpider. To confirm the presence of hyperoside, quercetin, kaempferol, and rutin, retention times (t_R_) and tandem MS (MS/MS) data were compared with those in the spectra of standards.

The mzCloud database contains MS and MS/MS spectra obtained on high-resolution mass spectrometers, such as Orbitrap Q Exactive HF, for various molecules. Such spectra can serve as a reference during a search for unknown substances. Both high-resolution data on whole molecular ions and all fragment ions are used for molecule identification. The Compound Discoverer software compares the registered whole molecular ion patterns and fragmentation patterns with those in the mzCloud database and scores possible variants. If there is a match, the program displays the identified compound with an assigned score from 0 to 100. The more the ions match, the higher the score is. In case of the absence of an analyzed molecular ion in the mzCloud database, a search was carried out in the ChemSpider database only by means of the exact mass and empirical formula. Therefore, such identification was more tentative. MS/MS spectra of the compounds tentatively identified by searching mzCloud and their comparison with mzCloud database spectra are presented in Table S1.

The flavonoids of *E. longistipitata* leaves turned out to be diverse. MS revealed 18 flavonoids: flavonols, flavones, chalcones, flavanones, and flavans. Flavonols featured the highest variety. Ten substances of this subfamily were identified. The identified flavonols are two aglycones, namely quercetin and kaempferol, and their O-glycosides, including six quercetin derivatives and two kaempferol derivatives. A substitution with hexose or pentose was found at the C-3 position, and both monosides (**2**, **3**, **7**, **8**, and **9**) and biosides (**1**, **5**, and **10**) were present (Figure 1). All the identified flavonols are rather common in the plant kingdom.

Carlinoside (**11**) is a flavone with an aglycone represented by luteolin. On ring A, sugar residues of glucose and arabinose are attached at positions C-6 and C-8 directly to the carbon atoms (meaning C-glycosylflavone; Figure 1). The concentration and type of C-glycosylflavones are often utilized as markers for identifying plants at the species level because of the high specificity of these compounds [20,21].

Cianidanol (**12**), which is better known as (+)-catechin, is 3,5,7,3′,4′-pentahydroxyflavan and is widely distributed in the plant kingdom [22]. Flavan auriculoside (**13**) has a similar structure but differs from cianidanol due to the absence of hydroxyls at positions C-3 and C-5 as well as the presence of a methoxy group at position 4′ and a glucose residue at position C-3′ of ring B (Figure 1). Auriculoside is the first flavan glucoside reported to have a depressant effect on the central nervous system [23,24].

6-Methoxytaxifolin (**14**) is known to be 12-fold sweeter than sucrose, and its 3-acetate is 25-fold sweeter than sucrose [25]. Aromadendrin (**15**), which is common in its free form and is often found in plant wood, has antitumor, anti-inflammatory, antidiabetic, and other activities [26,27].

In terms of the carbon skeleton, the identified chalcones are similar to phloridzin (**17**) but contain a glucose residue as a substituent at either the C-2′ or C-3′ position, and aspalathin (**16**) contains an additional hydroxyl group on ring B (Figure 1). Phloridzin and phloretin are chalcones common in the plant kingdom, and aspalathin is a rather rare C-dihydrochalcone, which was isolated from *Aspalathus linearis* (Burm.f.) R.Dahlgren in 1966. Until recently, aspalathin had been regarded only as a compound that is responsible for the characteristic color of Rooibos herbal tea; however, interest has shifted toward antioxidant properties of aspalathin and its beneficial influence on the metabolism of carbohydrates and lipids in humans [28,29].

Polyphenols play a pivotal role in structural integrity of plants, protection from UV radiation, reproduction, endogenous regulation of physiological processes, and plant cell signaling [30]. *E. longistipitata* begins to grow in late February/early March. In this period, insolation and spring frosts severely harm the plant. The plant’s tissues are protected from the damaging effects of external factors primarily by flavonoids, which inhibit free-radical reactions and directly participate in redox processes in plants. Flavonols play an important role in plants’ adaptation to temperature changes [31,32]. The observed diversity of flavonoids in the leaves of *E. longistipitata* is likely to be caused by external ecological and geographic factors.

**Figure 2:**
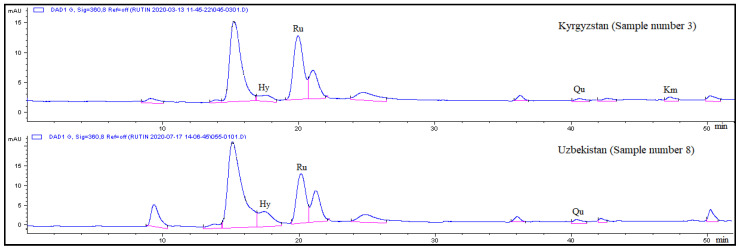
Chromatograms of 70%-aqueous-ethanol extracts from *E. longistipitata* leaves (detection at 360 nm). On the *X*-axis: retention time, min; on the *Y*-axis: the detector signal in units of optical density. Hy: hyperoside, Ru: rutin, Qu: quercetin, and Km: kaempferol.

Table 2 presents the levels of some flavonoids in the leaves of *E. longistipitata* from Kyrgyzstan (three samples) and Uzbekistan (five samples). Hyperoside, rutin, and quercetin were detectable in all the assayed samples, whereas kaempferol was absent in some leaf samples from Uzbekistan (Table 2 and Figure 2). Hyperoside concentrations in the leaves of *E. longistipitata* from Kyrgyzstan ranged from 0.79 to 1.01 mg/g, and in those from Uzbekistan, from 0.58 to 1.54 mg/g. The level of rutin in *E. longistipitata* leaves turned out to be the highest among all other identified compounds: 2.46 to 3.20 mg/g in the samples from Kyrgyzstan and 1.50 to 3.01 mg/g in the samples from Uzbekistan. Concentrations of quercetin (up to 0.62 mg/g) and kaempferol (up to 0.55 mg/g) are rather low in the aqueous-ethanol extracts from the leaves of the studied plants.

Aglycones of flavonols, quercetin, and kaempferol have been found in the leaves of *E. sibirica*, *E. stellata*, and *E. tanhoensis* by us previously [18]. Concentrations of these compounds are low and do not differ from those in the studied *E. longistipitata* and in other representatives of the genus *Eranthis*. Concentrations of quercetin glycosides, hyperoside and rutin in the leaves of *E. longistipitata* are higher than those of respective aglycones. These glycosides have strong pharmacological activities. For example, numerous in vitro and in vivo studies on the biological activity of hyperoside have shown that it exerts anti-inflammatory, antithrombotic, antidiabetic, antiviral, antifungal, hepatoprotective, and antioxidant protective effects [33]. Rutin is recommended for the treatment of various disorders, especially vascular diseases such as varicose veins, internal bleeding, or hemorrhoid. It is one of the most common quercetin glycosides and is found in a number of plants. The rutin level varies among different organs of plants from 0.148 to 620 mg/g. Buckwheat (*Fagopyrum esculentum* Moench) from the Polygonaceae family is the main natural source of rutin [34]. Concentrations of the identified flavonoids are not as high in the leaves of *E. longistipitata* as in widely recognized sources of these substances. Nevertheless, the combination of flavonoids in the aqueous-ethanol extract of this plant can have numerous effects that the polyphenols found in the leaves have.

## 3. Materials and Methods

### 3.1. Plant Material and Preparation of the Extract

Leaves of *E. longistipitata* were collected during the flowering–fruiting period in 2019 and 2020 (Table 3). Air-dried plant material was mechanically ground up to obtain a homogeneous powder. The flavonoids were studied in 70% aqueous-ethanol extracts of the leaves; these extracts were prepared by water bath extraction at 72 °C. A certain portion (0.200 g) of the crushed air-dried material was extracted twice: first, with 30 mL for 30 min, and then, with 20 mL for 20 min. After filtration, the residue in the flask and on the filter was washed with 5 mL of 70% ethyl alcohol. The mixed extract was then concentrated in porcelain cups down to 5 mL. Before the analysis, the solutions were filtered and stored at 4 °C.

### 3.2. MS Settings and the Spectral Library

LC-HRMS was carried out at the Core Facility of Mass Spectrometric Analysis at the Institute of Chemical Biology and Fundamental Medicine SB RAS (Novosibirsk, Russia). An Ultimate 3000 liquid chromatograph (Thermo Fisher Scientific, San Jose, CA, USA) coupled with a Q Exactive HF mass spectrometer (Thermo Fisher Scientific) was utilized to determine flavonoid profiles of *E. longistipitata* leaves. The chromatographic separation was attained at a 0.4 mL/min flow rate on a Zorbax Eclipse XDB-C18 reversed-phase column (150 × 3.0 mm, 5 μm, Agilent Technologies, Santa Clara, CA, USA) thermostated at 40 °C. The mobile phase was composed of 0.1% aqueous formic acid (eluent A) and acetonitrile (eluent B). The elution gradient was implemented as follows: from 5% to 70% B for 40 min, followed by an increase to 90% B for 8 min, a decrease to 5% B for 5 min, and re-equilibration under the initial conditions for 7 min.

The settings of the ESI source were as follows: electrospray voltage: 3.2 kV in the negative mode and 4.2 kV in the positive mode; capillary temperature: 320 °C; and the S lens RF level: 50. Data were obtained by full-scan data-dependent acquisition (FS-dd-MS2) in the positive and negative modes at resolving power of 45,000 full-width at half (FWHM) m/z 200. The following settings of the mass spectrometer were employed: scan range: m/z 80–1200; automatic gain control (AGC): 3e6; injection time: 100 ms; and the isolation window: m/z 2.0. The normalized collision energy for the fragmentation of molecular ions was set to 40 eV. A targeted MS/MS (dd-MS2) analysis was performed in both positive and negative modes at 15,000 FWHM (m/z 200). AGC for dd-MS2 was set to 1e5, with injection time of 50 ms and a loop count of 5. In the section of dd settings, the AGC target was programmed at 8e3, and maximum injection time was set to 100 ms. The data were analyzed using Xcalibur 4.0 and Compound Discoverer 3.1 software (Thermo Fisher Scientific). All the samples, including blank samples, were assayed in triplicate.

All the samples were processed in Compound Discoverer 3.1 via a common workflow called “Environmental Unknown ID w Online and Local Database Searches” (Figure S1). Mass tolerance of 5 ppm was applied to all nodes. Several databases, i.e., KEGG (https://www.genome.jp/kegg/; last accessed 10 March 2021), MassBank (https://massbank.eu/MassBank/; last accessed 10 March 2021), PlantCyc (https://plantcyc.org/; last accessed 10 March 2021), and Planta Piloto de Quimica Fina Universidad de Alcala (http://www.cqab.eu/index.php/en/; last accessed 10 March 2021), were chosen in the ChemSpider Search node of the workflow.

Flavonoids were identified on the basis of both accurate mass and fragment mass “fingerprint” spectra via searches against the spectra of compounds available in the mzCloud database (https://www.mzcloud.org; last accessed 10 March 2021). If compounds were absent in mzCloud, they were tentatively identified using a ChemSpider search. According to the workflow, the masses extracted from the chromatograms were aligned and filtered to remove i) background compounds present in the blank sample, ii) substances that failed to become fragmented, iii) compounds’ masses that were absent in the databases, and iv) signals with low intensity.

### 3.3. Flavonoid Analysis by High-Performance Liquid Chromatography

Quantification of flavonoids in the leaf samples was performed by means of an Agilent 1200 HPLC system equipped with a diode array detector and a ChemStation system for recording and processing chromatographic data (Agilent Technologies). The chromatographic separation was carried out on a Zorbax SB-C18 column (5 μm, 4.6 × 150 mm) at 25 °C. Methanol concentration in the mobile phase in an aqueous solution of phosphoric acid (0.1%) was increased from 50% to 52% for 56 min [35], and the eluent flow rate was 1 mL/min. Detection wavelengths were 254, 270, 290, 340, 360, and 370 nm. Individual compounds were quantified by an external standard method [29]. Mean values are expressed in milligrams per gram of air-dried matter.

### 3.4. Chemicals

All chemicals were of MS or analytical grade. Chemical reference standards of quercetin, kaempferol, and rutin were purchased from Sigma-Aldrich (Germany). Rutin and chemical reference standards of hyperoside were purchased from Fluka Chemie AG (Switzerland).

### 3.5. Statistical Analysis

All samples, including blank samples, which consisted of the pure solvent, were analyzed as two biological replicates with three technical replicates per treatment group. Data are expressed as means ± SD if not stated otherwise. Multiple comparisons were performed using one-way ANOVA followed by Tukey’s honest significant difference test to evaluate the significance of differences among means. Differences were assumed to be significant at *p* < 0.05.

## 4. Conclusions

The rich flavonoid profile of *E. longistipitata* leaves includes flavonols, flavones, flavans, flavanones, and chalcones. The highest diversity was shown by flavonols. The concentration of rutin reaches 3.20 mg/g in the aqueous-ethanol extracts from leaves of the studied plants from natural populations in Kyrgyzstan and Uzbekistan. In the future, it will be possible to test *Eranthis* plants for the possible biological activities that are characteristic of the identified subfamilies of flavonoids.

## Figures and Tables

**Table 1:** Flavonoids identified in aqueous-ethanol extracts from *E. longistipitata* leaves by LC-HRMS using databases mzCloud and ChemSpider.

ID	t_R_ (min)	Calculated Mass	Measured Mass	Delta Mass [Da]	Delta Mass [ppm]	MzCloud Score	Mode	Identified Compounds
Flavonols								
1	10.13	596.13773	596.13689	−0.00084	−1.42	‒	Positive	Peltatoside (quercetin-3-(6-*O*-α-L- arabinopyranosyl)-β-D- glucopyranoside))
2	10.95	464.09548	464.09524	−0.00024	−0.51	98.2	Positive	Hyperoside * (quercetin 3-*O-*β-D- galactoside)
3	10.95	434.08491	434.08460	−0.00031	−0.72	98.7	Positive	Reynoutrin (quercetin-3-*O*-β-D- xylopyranoside)
4	11.29	302.04265	302.04231	−0.00035	−1.15	99.9	Positive	Quercetin *
5	11.66	596.13773	596.13714	−0.00060	−1.00	98.1	Negative	Quercetin 3-sambubioside (quercetin-3-*O*-[β-D-xylosyl- (1→2)-β-D-glucoside])
6	11.90	286.04774	286.04751	−0.00023	−0.80	99.0	Positive	Kaempferol *
7	11.90	418.09000	418.08966	−0.00033	−0.79	79.8	Positive	Juglalin (kaempferol 3-*O*-α-L- arabinopyranoside)
8	11.90	448.10056	448.10032	−0.00024	−0.54	98.5	Positive	Trifolin (kaempferol-3-*O*-β-D- galactoside)
9	13.01	464.09548	464.09582	0.00035	0.75	99.2	Negative	Isoquercitrin (quercetin-3-*O*-β-D- glucoside)
10	12.48	610.15338	610.15249	−0.00089	−1.46	98.9	Positive	Rutin * (quercetin 3-*O*-β-D- rutinoside)
Flavones								
11	12.56	580.14282	580.14259	−0.00023	−0.40	‒	Negative	Carlinoside (luteolin 6-*C*-β-D- glucopyranoside-8-*C*-α-L- arabinopyranoside)
Flavans								
12	16.53	290.07904	290.07898	−0.00003	−0.11	‒	Positive	Cianidanol ((+)-catechin)
13	19.78	450.15260	450.15215	−0.00045	−0.99	‒	Positive	Auriculoside (7,3′,5′-trihydroxy-4′-methoxyflavan-3′-glucoside)
Flavanones								
14	14.55	334.06887	334.06903	0.00016	0.48	‒	Negative	6-Methoxytaxifolin
15	21.50	288.06339	288.06336	−0.00003	−0.11	‒	Positive	Aromadendrin ((+)-dihydrokaempferol)
Chalcones								
16	15.45	452.13186	452.13172	−0.00014	−0.32	‒	Positive	Aspalathin
17	16.23	436.13695	436.13681	−0.00013	−0.30	‒	Positive	Phloridzin (phloretin-2′-*O*-β-glucoside)
18	20.80	274.08412	274.08393	−0.00019	−0.69	‒	Positive	Phloretin (dihydroxy naringenin)

Note: * compounds confirmed by means of standards; “‒”: only ChemSpider.

**Table 2:** Levels of some identified flavonoids in *E. longistipitata* leaves (mg/g of air-dried matter).

Sample No.	Hyperoside (t_R_ = 17.5 min)	Rutin (t_R_ = 20.1 min)	Quercetin (t_R_ = 40.6 min)	Kaempferol (t_R_ = 47.0 min)
1	1.01 ± 0.04 ^c^	2.49 ± 0.09 ^b^	0.62 ± 0.02 ^a^	0.55 ± 0.02 ^a^
2	0.88 ± 0.03 ^d^	2.46 ± 0.09 ^b^	0.12 ± 0.00 ^g^	0.20 ± 0.01 ^c^
3	0.79 ± 0.03 ^d^	3.20 ± 0.12 ^a^	0.27 ± 0.01 ^ef^	0.28 ± 0.01 ^b^
4	0.97 ± 0.04 ^c^	2.09 ± 0.08 ^c^	0.41 ± 0.02 ^c^	–
5	1.33 ± 0.05 ^b^	3.01 ± 0.11 ^a^	0.55 ± 0.02 ^b^	–
6	0.58 ± 0.02 ^e^	1.50 ± 0.06 ^d^	0.28 ± 0.01 ^e^	0.10 ± 0.00 ^e^
7	1.54 ± 0.06 ^a^	2.28 ± 0.08 ^bc^	0.23 ± 0.01 ^f^	0.13 ± 0.00 ^d^
8	1.49 ± 0.06 ^a^	2.04 ± 0.08 ^c^	0.33 ± 0.01 ^d^	–

Note: The data are presented as means and standard error (*n* = 3). Data followed by different lowercase letters (a–g) in the same row are significantly different (*p* ≤ 0.05) according to Tukey’s honest significant difference test; “–“: not found.

**Table 3:** Sites of collection of the analyzed samples.

Sample No.	Locality; Coordinates	Habitat	Date
1	Kyrgyzstan, Chuya region, Issyk-atinskii district, Niczniaya Serafimovka village; 42°45′02″ N, 74°51′37″ E	foot of the mount	19.03.2019
2	Kyrgyzstan, Chuya region, Issyk-atinskii district, Karandolot tract; 42°44′22″ N, 74°55′50″ E	foot of the mount	22.03.2019
3	Kyrgyzstan, Talas region, Kara-Buurinskii district, west of Kirovskoe reservoir; 42°37′57″ N, 71°34′47″ E	steppe	26.03.2019
4	Uzbekistan, Andijan region, Khojaabad district, east-southeastern part of Fergana valley, Kyrtashtau mountains, near Imamat village; 40°32′27″ N, 72°36′28″ E	mossy stony slope	12.03.2020
5	Uzbekistan, Samarkand region, Urgut district, western Pamir-Alai, Gissar-Alai, western part of the Zeravshan ridge, right bank of Amankutansai river, near Amankutan kishlak; 39°18′16″ N, 66°55′45″ E	juniper forest on the slope	14.03.2020
6	Uzbekistan, Tashkent region, Bostanlyk district, western Tian Shan, spurs of northwestern part of Chatkal ridge, Galvasay river valley—left tributary Chirchik river, left bank; 41°32′20″ N, 69°53′03″ E	walnut grove on the slope	16.03.2020
7	Uzbekistan, Tashkent region, Bostanlyk district, Western Tian Shan, north-western part of Chatkal ridge, foot of Big Chimgan mountain, area between Galvasay and Mramornaya rivers, on road from Uchterek tract to Chimgan tract; 41°31′05″ N, 69°59′15″ E	bushy slope	16.03.2020
8	Uzbekistan, Jizzakh region, Zaamin district, western Pariro-Alai, Gissar-Alai, northern macroslope of Turkestan ridge, Zaamin forestry enterprise, Usman tract, 39°43′26″ N, 68°27′54″ E	mountain slope	20.03.2020

## Data Availability

Not applicable.

## Supplementary Materials

The following are available online at https://www.mdpi.com/article/10.3390/plants10102146/s1: Figure S1: The workflow on Compound Discoverer used for flavonoid identification, and Table S1: Comparison of the obtained spectra with the spectra of standards available in the mzCloud database.
