# Supplementary material for: Identification of Flavonoids in the Leaves of Eranthis longistipitata (Ranunculaceae) by Liquid Chromatography with High-Resolution Mass Spectrometry (LC-HRMS)
Source: Plants (Basel). 2021 Oct 10;10(10):2146. doi: 10.3390/plants10102146 (PMC8539099; doi:10.3390/plants10102146)
Supplement: Supplementary file 1 [file plants-10-02146-s001.zip › plants-1402294-supplementary.pdf]

Supplementary Material

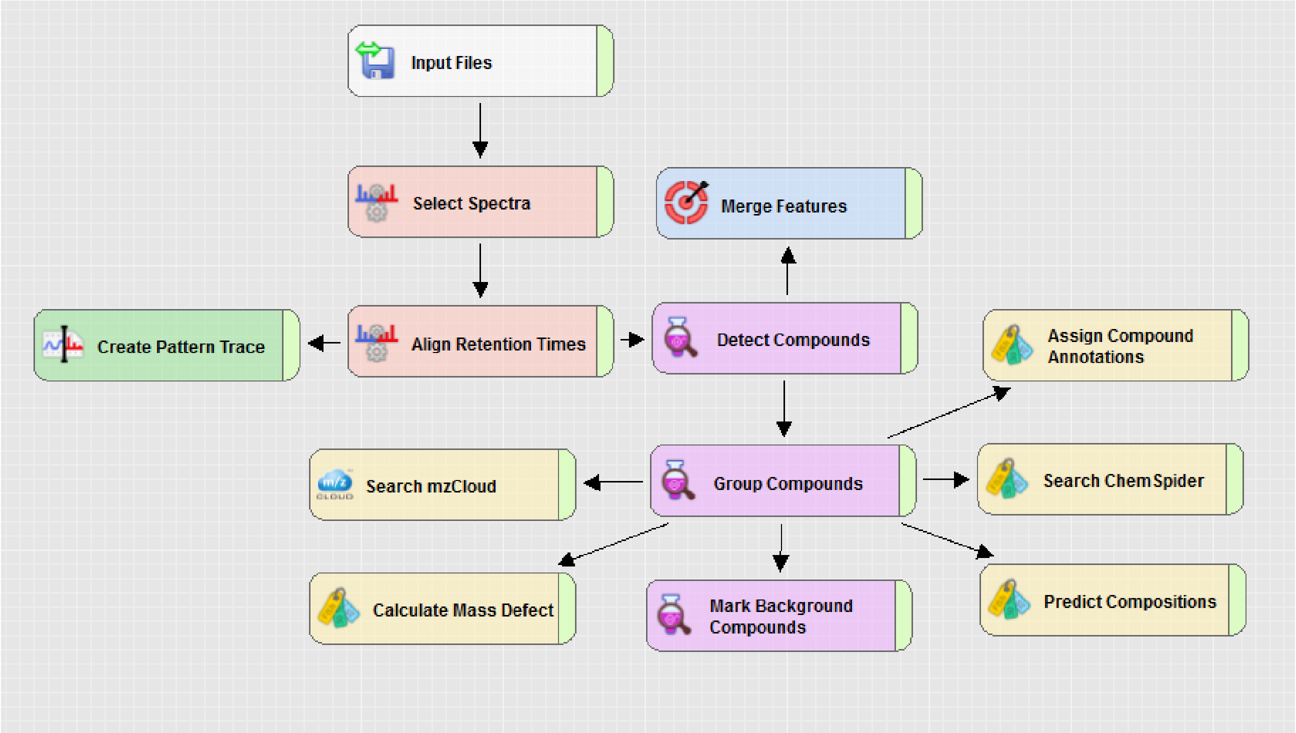

Figure S1. The workflow on Compound Discoverer used for flavonoid identification

Table S1. Comparison of the obtained spectra with the spectra of standards available in the mzCloud database.

<table>
<tr><th>ID</th><th>Measured MS/MS (upper)vs mzCloud MS/MS (down)</th><th>Identified compounds</th></tr>
<tr><td>2</td><td>
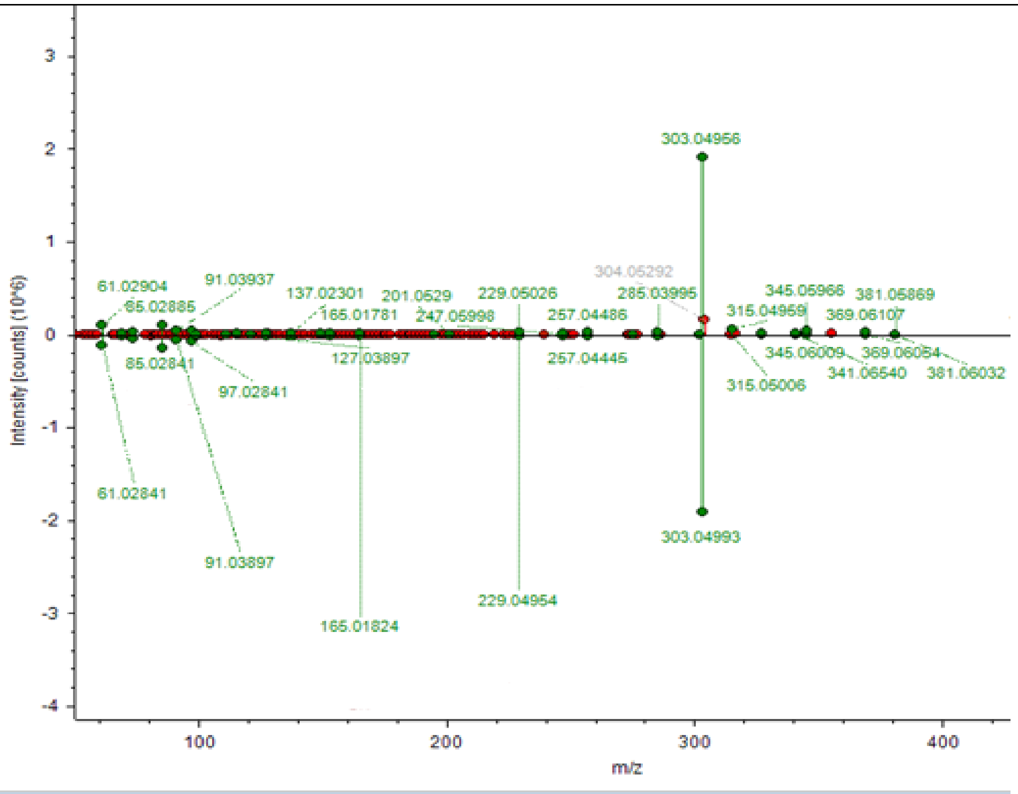

</td><td>Hyperoside* (quercetin 3-O-β-D-galactoside)</td></tr>
<tr><td>3</td><td>
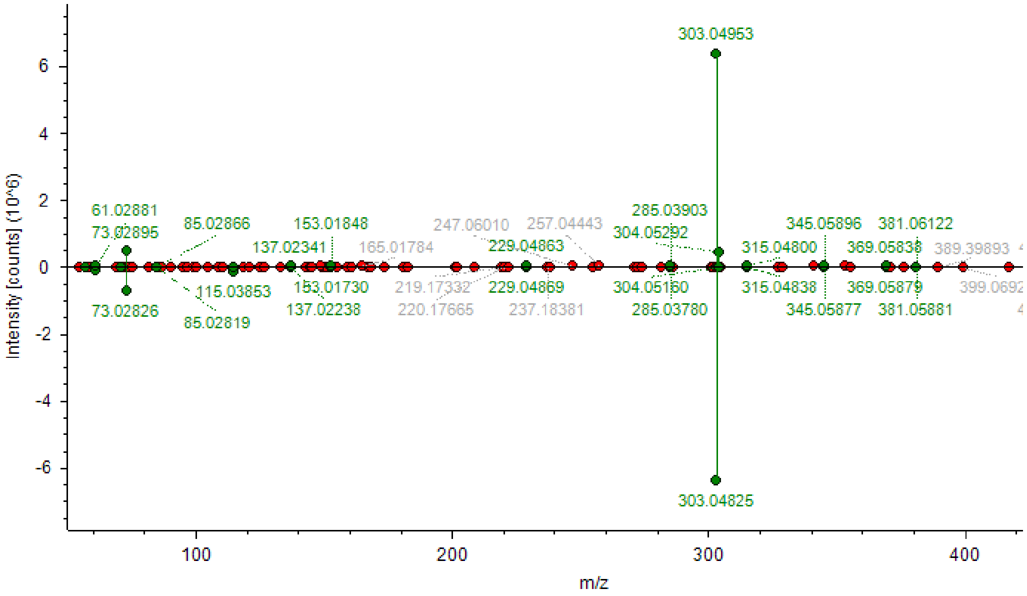

</td><td>Reynoutrin (quercetin-3-O-β-D-xylopyranoside)</td></tr>
</table>

<table>
<tr>
<td>4</td>
<td>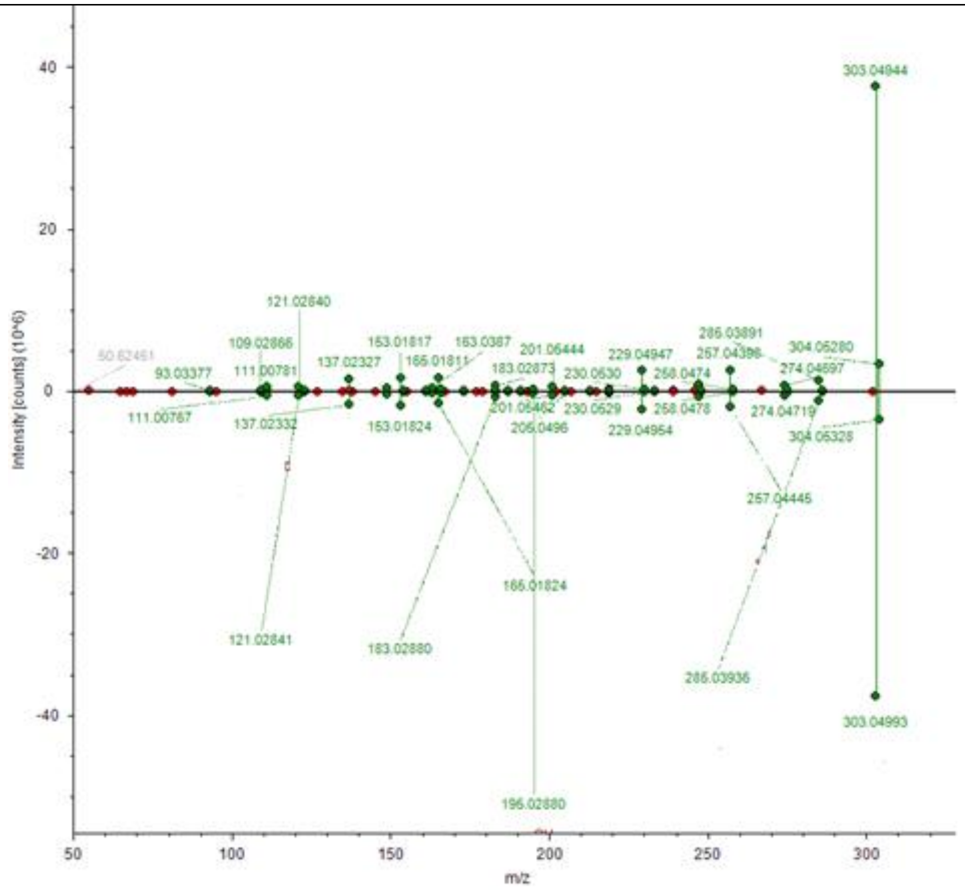
</td>
<td>Quercetin*</td>
</tr>
<tr>
<td>5</td>
<td>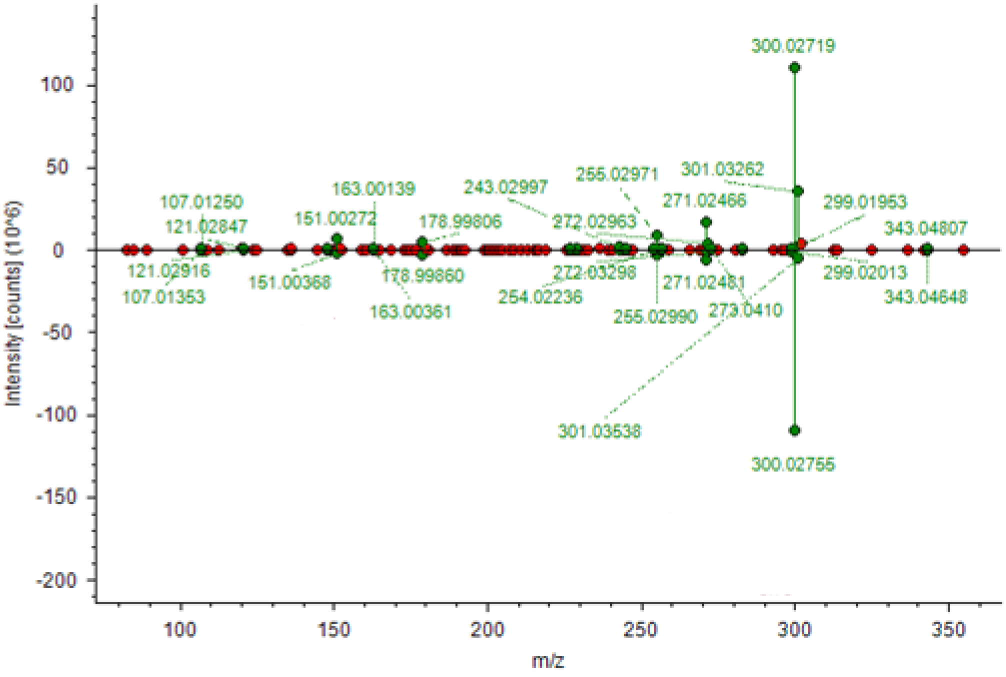
</td>
<td>Quercetin 3-sambubioside (quercetin-3-O-[β-D-xylosyl-(1→2)-β-D-glucoside])</td>
</tr>
</table>

6	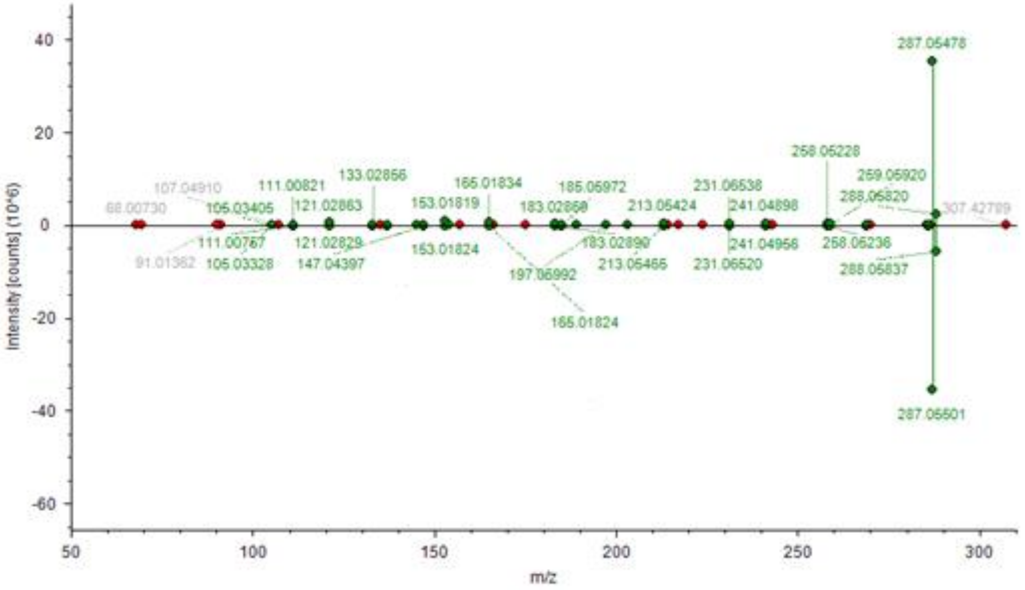	Kaempferol*
7	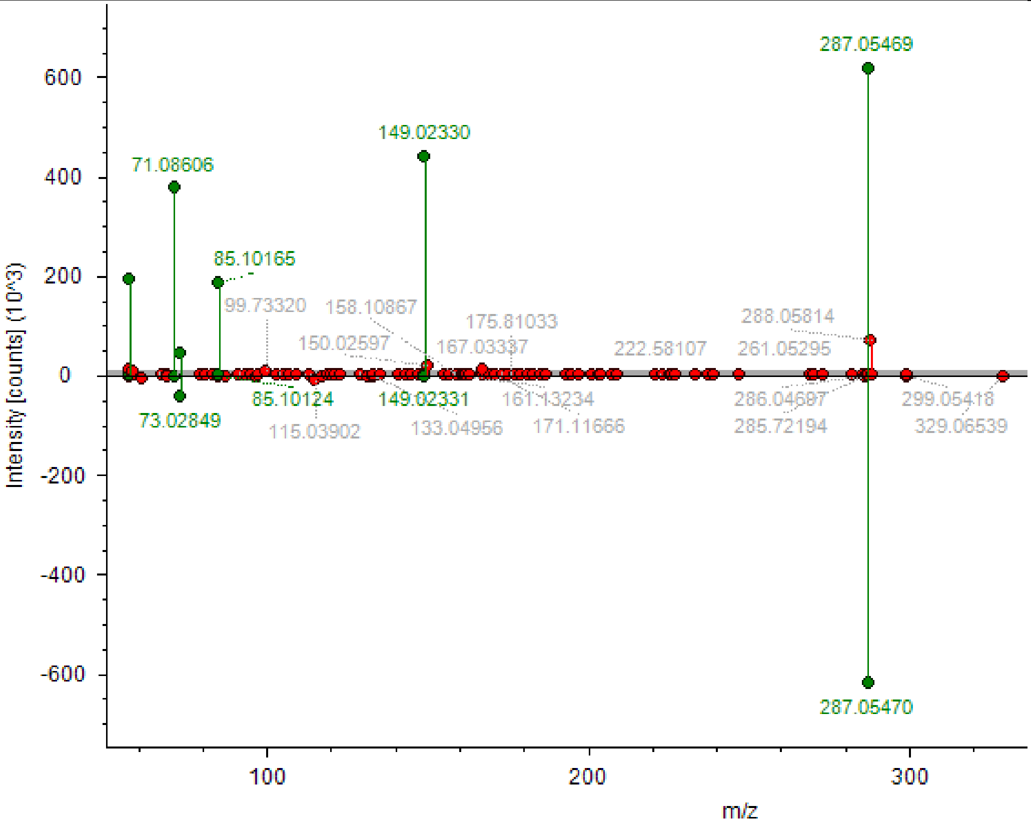	Juglalin (kaempferol 3-*O*-α-L-arabinopyranoside)

8	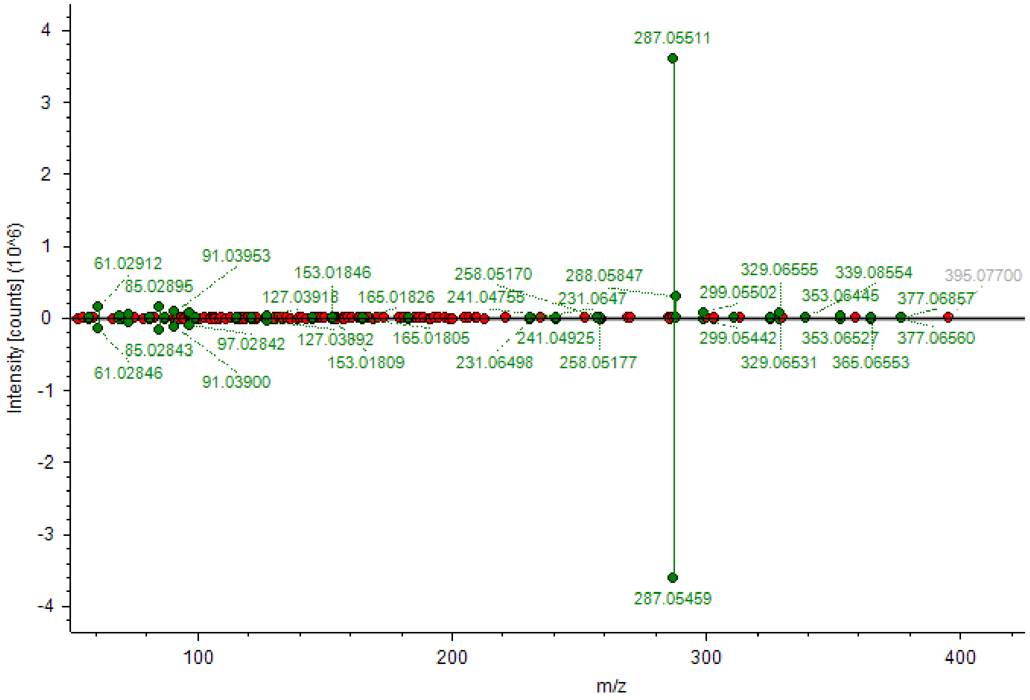	Trifolin (kaempferol-3-O-β-D-galactoside)
9	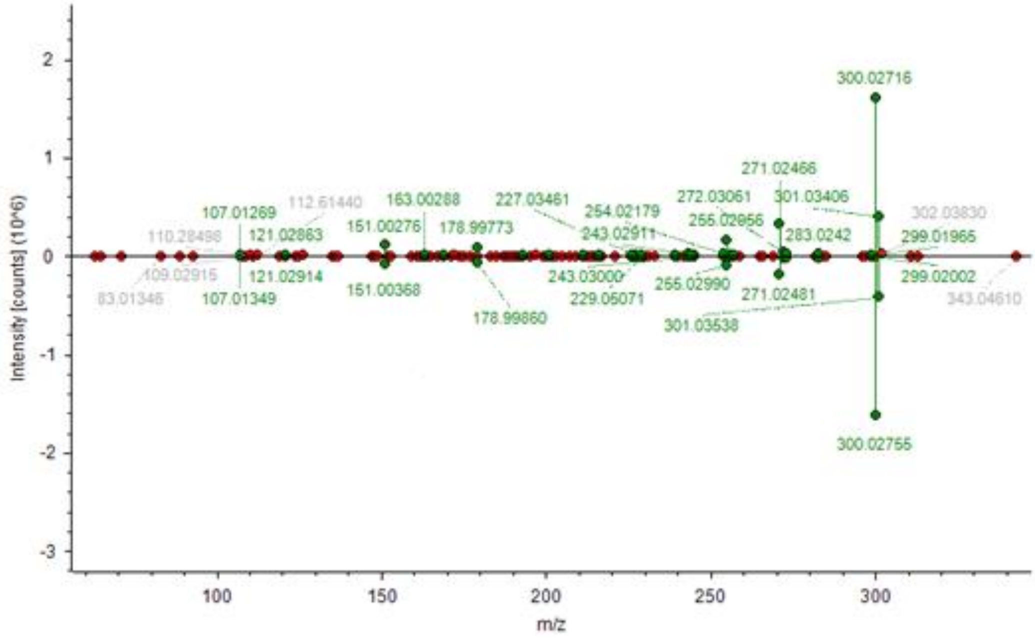	Isoquercitrin (quercetin-3-*O*-β-D-glucoside)

<table>
<tr>
<td>10</td>
<td>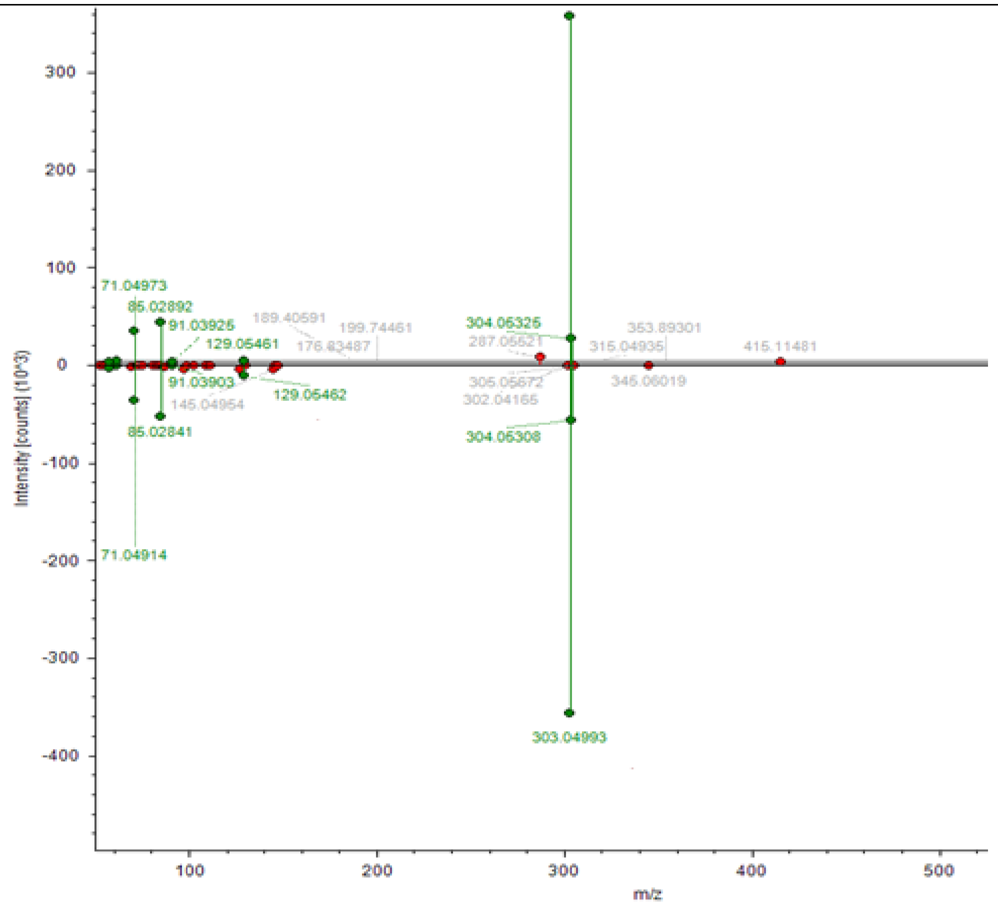
</td>
<td>Rutin*

(quercetin 3-O-β-D-rutinoside)</td>
</tr>
</table>

The same fragments in the measured spectrum and in the mzCloud database are colored by green, the mismatch fragments are colored by red.
